# Intense second-harmonic generation in two-dimensional PtSe_2_

**DOI:** 10.1515/nanoph-2024-0107

**Published:** 2024-04-22

**Authors:** Lingrui Chu, Ziqi Li, Han Zhu, Hengyue Lv, Feng Chen

**Affiliations:** School of Physics, State Key Laboratory of Crystal Materials, 12589 Shandong University, Jinan 250100, China; Division of Physics and Applied Physics, School of Physical and Mathematical Sciences, 54761 Nanyang Technological University, Singapore 637371, Singapore

**Keywords:** platinum diselenide (PtSe_2_), second-harmonic generation, nonlinear optics

## Abstract

Platinum diselenide (PtSe_2_), classified as a noble metal dichalcogenide, has garnered substantial interest owing to its layer-dependent band structure, remarkable air-stability, and high charge-carrier mobilities. These properties make it highly promising for a wide array of applications in next-generation electronic and optoelectronic devices, as well as sensors. Additionally, two-dimensional (2D) PtSe_2_ demonstrates significant potential as a saturable absorber due to its exceptional nonlinear optical response across an ultrabroad spectra range, presenting exciting opportunities in ultrafast and nonlinear photonics. In this work, we explore the second-order nonlinear optical characteristics of 2D PtSe_2_ by analyzing its second-harmonic generation (SHG) excited by a pulsed laser at 1064 nm. Our investigation unveils a layer-dependent SHG response in PtSe_2_, with prominent SHG intensity observed in few-layer PtSe_2_. The distinct six-fold polarization dependence pattern observed in the SHG intensity reflects the inherent threefold rotational symmetry inherent to the PtSe_2_ crystal structure. Remarkably, the SHG intensity of 4-layer PtSe_2_ surpasses that of mechanically exfoliated monolayer molybdenum disulfide (MoS_2_) by approximately two orders of magnitude (60-fold), underscoring its exceptional second-order nonlinear optical response. Combined with its ultrahigh air-stability, these distinctive nonlinear optical characteristics position two-dimensional PtSe_2_ as a promising candidate for ultrathin nonlinear nanophotonic devices.

## 1 Introduction

Second-harmonic generation (SHG) in two-dimensional (2D) materials is an intriguing optical phenomenon where electrons are confined into a two-dimensional plane, leading to unique nonlinear optical behavior. When exposed to incident light, the electrons undergo oscillations, emitting light waves at twice the frequency through SHG process. This effect is particularly significant due to the exceptional electronic and optical properties of 2D materials, attracting considerable attention in nonlinear photonics and quantum technologies [1], [2], [3], [4], [5], [6]. The SHG process in 2D materials is intricately linked to their electronic structure and crystal symmetry [7]. Materials with centrosymmetric properties typically lack SHG effects because their second-order nonlinear optical susceptibility is vanishing [8]. Conversely, non-centrosymmetric 2D materials, like molybdenum disulfide (MoS_2_) monolayer, exhibit strong SHG signals owing to its symmetry breaking [9], [10], [11]. Strong SHG signal has also been observed in other 2D materials, like WS_2_ [12], WSe_2_ [13], ReS_2_ [14], GaSe [15], [16], making these 2D materials attractive for the development of ultrathin nonlinear optical devices.

Manipulating external parameters, such as strain, electric field, incident light intensity, and polarization, offers precise control over SHG in 2D materials [17], [18], [19], [20], [21], [22], [23]. For example, strain engineering can alter the lattice structure of graphene, inducing sublattice polarization to enhance SHG in monolayer graphene [17]. External electric fields can induce strong, doping-induced SHG in centrosymmetric monolayer graphene, comparable in strength to SHG in non-centrosymmetric 2D materials [20]. Additionally, electrostatic doping can tune the intensity of SHG at the A-exciton resonance of WSe_2_ by over an order of magnitude at low temperatures and nearly fourfold at room temperature in a field-effect transistor [19]. This level of control offers exciting prospects for applications, including high-efficiency nonlinear devices and electrically tunable nonlinear optical devices for integrated quantum circuits.

Understanding the underlying principles which govern SHG in these materials is paramount for the design and development of novel devices with enhanced performance and functionality. By finely tailoring the characteristics of two-dimensional (2D) materials, it becomes possible to enhance and manipulate the efficiency of SHG and their nonlinear optical response [8], [24], [25]. For instance, the chemical vapor deposition (CVD)-grown spiral tungsten disulfide (WS_2_) nanosheets demonstrate a stronger SHG intensity that quadratically increases with their layer numbers due to the broken symmetry resulting from twisted screw structures [8]. This phenomenon extends the potential applications of 2D materials in nonlinear optical technologies. The exploration of novel materials with distinct electronic or optical characteristics has led to the investigation of 2D materials exhibiting high-efficiency nonlinear optical responses for next-generation photonic technologies [3], [26], [27]. One such material, layered niobium oxide dichloride (NbOCl_2_), possesses vanishing interlayer electronic coupling and monolayer-like excitonic behavior even in bulk form. Notably, NbOCl_2_ exhibits a scalable SHG intensity of up to three orders higher than that observed in conventional monolayer WS_2_, rendering it highly attractive for integration into nonlinear nanophotonic devices. Moreover, its strong second-order nonlinearity enables correlated parametric photon pair generation through a spontaneous parametric down-conversion (SPDC) process, even in flakes as thin as approximately 46 nm, establishing it as the thinnest SPDC source ever reported [3].

Beyond its potential applications in photonics, understanding the intricacies of SHG in 2D materials could lead to breakthroughs in materials science [28], [29] and fundamental physics [30]. For instance, SHG in 2D materials is a highly sensitive probe of subtle magnetic orders, the observed giant nonreciprocal SHG revealing the layered antiferromagnetic order in chromium triiodide (CrI_3_), opening up possibilities for the use of two-dimensional magnets in nonlinear and nonreciprocal optical devices, holding immense potential for unlocking unprecedented opportunities in next-generation photonic technologies [26], [31]; Insights gained from investigating SHG could also shed light on the underlying electronic structure and symmetry properties of 2D materials, advancing our knowledge of condensed matter physics [7], [29], [32], [33].

As representatives of 2D materials, graphene and conventional transition metal dichalcogenides like MoS_2_, MoSe_2_ have captured considerable attention over the past decades [34], [35], [36]. Beyond these established materials, a new class of layered materials has emerged, each offering unique functionalities. Among them, black phosphorus has gained prominence for its distinctive properties for its ultra-high electron mobility [37], [38]. Another notable 2D material is the class of noble metal dichalcogenides (NMDs) [39], [40], [41], [42], [43]. Among them, compounds such as PtSe_2_ and PdSe_2_ have recently garnered significant interest owing to their remarkable characteristics. These materials exhibit exceptional environmental stability, outstanding electronic properties [41], [42], and giant optical nonlinearity [44].

Notably, layered PtSe_2_, with its capacity for low-temperature synthesis, coupled with its high charge-carrier mobilities and enduring air stability, underscores its potential for electronic and optoelectronic applications [40], [45], [46]. For instance, devices composed of a few layers of PtSe_2_ demonstrate semiconducting behavior with impressive environmental resilience, showcasing a noteworthy room-temperature electron mobility of 210 cm^2^ V^−1^ s^−1^ when configured with a back gate on a SiO_2_/Si substrate [47]. Furthermore, bilayer PtSe_2_, when combined with defect modulation, exhibits strong light absorption in the mid-infrared region, enabling the realization of a broadband mid-infrared photoconductive detector [46]. In addition, thin polycrystalline films of PtSe_2_, a centrosymmetric Dirac semimetal, exhibit giant photon momentum-locked terahertz (THz) emission, presenting exciting prospects for highly efficient THz signal activation [48]. While the nonlinear optical absorption of layered PtSe_2_ has been extensively studied [49], [50], its second-order optical nonlinearity remains unexplored. By measuring and analyzing the SHG signal of layered PtSe_2_, its second-order optical nonlinear properties could be further explored to uncover potential applications in nonlinear optics and beyond.

In this study, we explore the second-order optical nonlinearity of layered PtSe_2_ through the characterization of its SHG excited by a pulsed laser operating at 1064 nm. The layered PtSe_2_ samples are firstly prepared via gold-assisted mechanical exfoliation, ensuring their high quality. The SHG measurements reveal that few-layer PtSe_2_ exhibits a pronounced SHG intensity when pumped at 1064 nm. The inherent threefold rotation symmetry of the PtSe_2_ crystal manifests in a distinct six-fold polarization dependence pattern observed in the SHG intensity. Notably, we observe a remarkable two-orders-of-magnitude enhancement (60-fold) in the SHG intensity of 4-layer PtSe_2_ compared to conventional monolayer MoS_2_, revealing the significant second-order optical nonlinear response exhibited by 2D PtSe_2_. The strong SHG exhibited by 2D PtSe_2_, especially in the case of 4-layer PtSe_2_, positions 2D PtSe_2_ as an attractive option for ultrathin and efficient nonlinear optical devices.

## 2 Results and discussion

**Figure 1:**
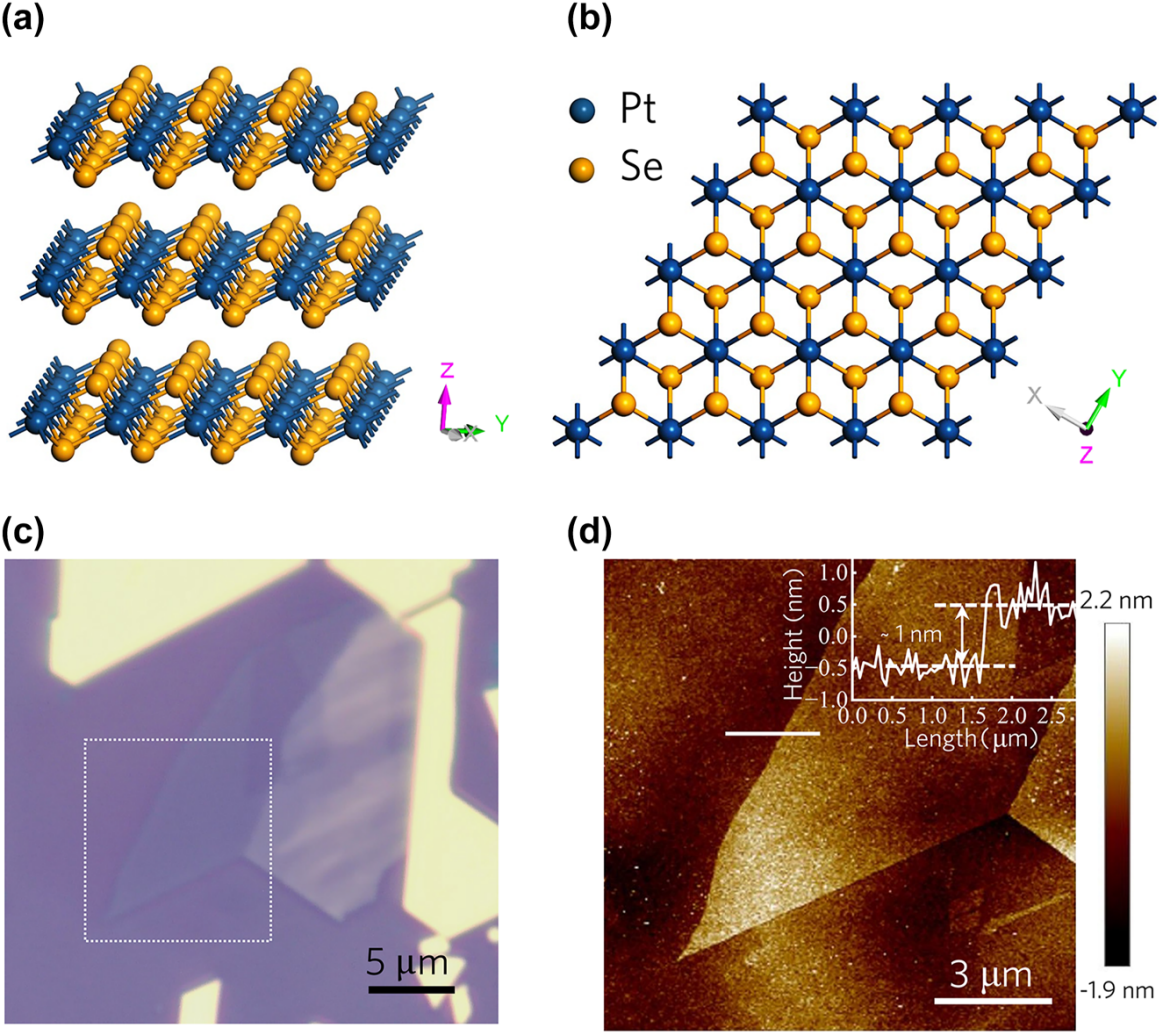
Crystallographic structure and AFM characerization of layered-PtSe_2_. (a) and (b) Indicate the lattice structure of the layered PtSe_2_. Blue and yellow globes denote the Pt and Se atoms, respectively. (c) The optical microscope image of gold-assisted mechanically exfoliated few-layer PtSe_2_. (d) The AFM image of layered PtSe_2_ in box region denoted in (c). The inset demonstrates a measured height of ∼1 nm, which corresponds to the thickness of bilayer PtSe_2_.

Figure 1(a) and (b) present the crystal structure of van der Waals PtSe_2_, illustrating its distinctive atomic arrangement characterized by layers of atoms arranged in a typical 1*T*-type hexagonal crystal structure with the *P3m1* space group. Each layer consists of one platinum atom sandwiched between two selenium atoms, tightly bonded through covalent interactions within the layer, while weak Van Der Waals forces maintain cohesion between adjacent layers.

In contrast to conventional transition metal dichalcogenides (TMDs), PtSe_2_ demonstrates significantly stronger interlayer interactions, as evidenced by its calculated interlayer energy of 0.342 eV per unit cell [51]. Consequently, the mechanical exfoliation of layered PtSe_2_ flakes using conventional methods proves challenging. To address this, the developed gold-assisted mechanical exfoliation method [51], [52], [53] is employed to exfoliate a PtSe_2_ single-crystal (purchased from 2D Semiconductors) into layered PtSe_2_ flakes. In layered materials, the interaction between layers is primarily through van der Waals forces, while Au and many 2D materials including PtSe_2_ can form covalent bonds. This interaction is stronger than van der Waals forces but weaker than covalent bonds, allowing for efficient exfoliation of large-area single-layer samples without affecting the intrinsic properties of the material. Initially, a Au (4 nm)/Ti (2 nm) thin film is deposited onto a silicon substrate at an evaporation rate of 0.5 Å/s via electron beam evaporation (utilizing the HHV Auto500). The 2 nm Ti layer served as an adhesion layer. Subsequently, the freshly cleaved layered bulk PtSe_2_ crystal on tape is promptly brought into contact with the upper Au layer. Upon removal of the tape, the predominant portion of the crystal is extracted, leaving behind a few two-dimensional PtSe_2_ layer flakes on the Au surface, successfully preparing the 2D layered PtSe_2_.

The optical microscope image of the fabricated layered PtSe_2_ on the SiO_2_/Si substrate is captured using a microscope equipped with a CCD (ZEISS, Axiocam 208 color). The thickness of the layered PtSe_2_ is characterized by an atomic force microscope (Bruker, BioScope Resolve). Figure 1(c) displays an optical microscope image of the exfoliated 2D PtSe_2_ on a silicon substrate. Lighter colors indicate thinner layers, with the white dashed box region highlighting atomic-thin PtSe_2_ flakes, while the yellow portions represent bulk material. The layer numbers could be characterized by the atomic force microscopy (AFM) measurements. AFM characterization of the thinner region within the box, as shown in Figure 1(d), reveals the atomic-thickness nature of the exfoliated flakes. The height profile along the white line denoted in Figure 1(d) confirms a thickness of approximately 1 nm, corresponding to bilayer PtSe_2_ thickness with a single layer thickness of 0.5 nm (inset of Figure 1(d)). Furthermore, Raman spectra are acquired to further characterize the exfoliated PtSe_2_ flakes, confirming the quality of the prepared sample (Figure S1).

**Figure 2:**
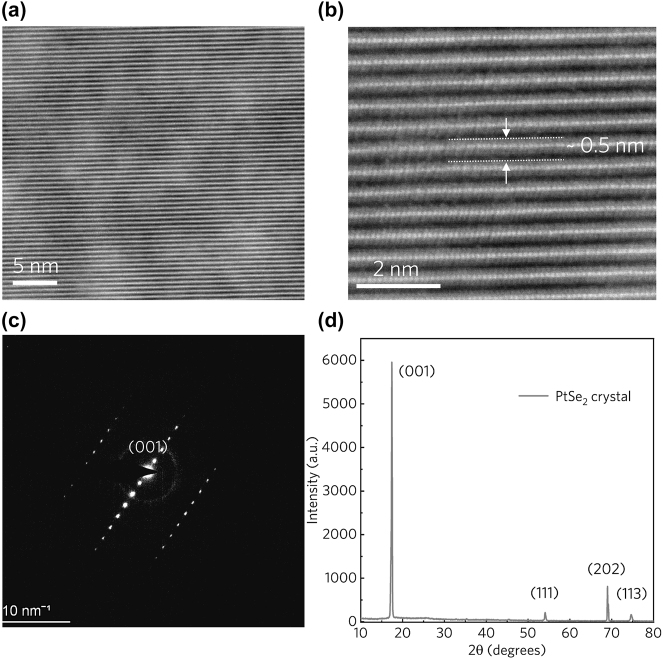
STEM and XRD characterization of layered PtSe_2_. (a) STEM image of the layered PtSe_2_ flakes. (b) High-magnification STEM image of layered PtSe_2_. The Pt and Se atoms could be clearly observed, every layer consists of one Pt atom sandwiched between two Se atoms. (c) SAED pattern of the PtSe_2_. (d) XRD analysis of PtSe_2_ crystal. The diffraction pattern exhibits characteristic (001), (111), (202), (113), which is corresponding to hexagonal crystal phase (JCPDS PDF#: 00-065-3374).

Scanning transmission electron microscopy (STEM) images (measured by Thermo Scientific Spectra 300) and X-ray diffraction (XRD) patterns (obtained by Smartlab9KW) are employed to elucidate atomic structure and crystal quality of the prepared layered PtSe_2_. Figure 2(a) presents a cross-sectional STEM image of a thin PtSe_2_ flake, revealing the distribution of Se and Pt atoms in the side view. The absence of obvious vacancies confirms the high quality of the PtSe_2_ crystal. The observed layer pitch of approximately 0.5 nm aligns well with previous experimental findings (Figure 2(b)).

As shown in Figure 2(c), Analysis of selected area electron diffraction (SAED) patterns confirms the single-crystalline nature of PtSe_2_, with a clear identification of the (001) crystal orientation. Furthermore, XRD analysis provides further insights into the crystal structure. The presence of four major characteristic peaks indicates the crystal’s high quality, with other peaks likely suppressed due to the layered structure and specific (001) orientation of PtSe_2_ (Figure 2(d)). Additionally, the presence of strong, narrow peaks corresponding to (001), (111), (202) and (113) lattice planes underscores the exceptional crystal quality of the fabricated samples [46], [54].

The SHG measurements are conducted using a microscope employing vertical reflection configuration. A pulsed laser, with a pulse width approximately 10 ps and a repetition rate around 20 MHz, emitting, at the central wavelength of 1064 nm, is precisely focused onto the sample at a normal angle of incidence through a 100× objective lens. The SHG signal, reflected from the sample, is gathered by the same objective lens and subsequently captured by a grating spectrometer fitted with a CCD camera. In the experiments, polarization-dependent SHG measurement is performed by placing a half-wave plate and analyzer in the incident and detection paths, respectively. In the parallel configuration, a step size of 10° is used. Through SHG mappings, the layer-dependent SHG intensity could be clearly distinguished. The layer-dependent SHG intensity could be clearly distinguished by conducting SHG mappings with a laser spot size of 0.5 μm and high-resolution step size of 0.3 μm. The schematic diagram of the experimental setup can be found in Figure S2 in the Supplementary Material.

**Figure 3:**
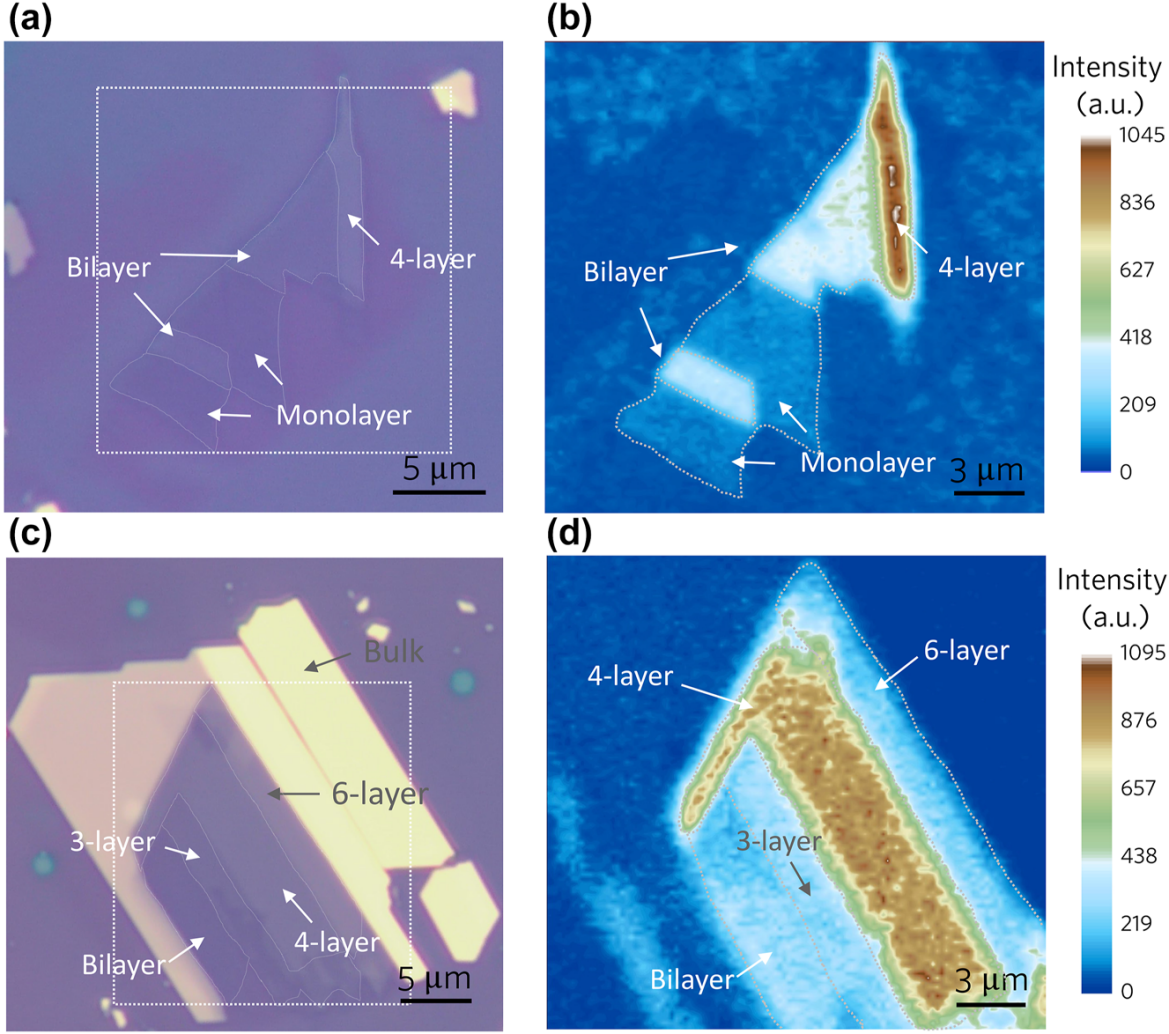
The optical microscope image of exfoliated layered PtSe_2_ and corresponding SHG mapping figures. (a) The optical microscope image of the layered PtSe_2_ including monolayer, bilayer, and 4-layer PtSe_2_, as indicated by the white arrow. (b) The SHG mapping of the box region. The 4-layer PtSe_2_ exhibits a strong SHG intensity, while the monolayer PtSe_2_ exhibits a weak SHG response. (c) The optical microscope image of layered PtSe_2_ consisting of bilayer, 3-layer, 4-layer, and 6-layer PtSe_2_. (d) The SHG mapping of the box region in (c). The 4-layer PtSe_2_ exhibits a relatively strong intensity.

By comparing Figure 3(a) and (b), we observe a very weak SHG signal in monolayers. The lattice structures of 1*T*-type PtSe_2_ belongs to D_3d_ with preserved centrosymmetry and no intense SHG is expected to occur in layered PtSe_2_. Interestingly, intense SHG is observed for few-layer PtSe_2_. Similar phenomenon has been reported in 1*T*-type TiSe_2_ and SnSe_2_ [55], [56]. One of its possible origins is the occurred lattice distortion associated with the charge density waves [56]. Compared to bilayer PtSe_2_, we can observe a stronger signal in 4-layer PtSe_2_. Therefore, we further conducted SHG mapping tests on exfoliated few-layer PtSe_2_ samples. As shown in Figure 3(c) and (d), we can see that the SHG signal intensity does not increase linearly with the number of layers. Instead, the strongest SHG signal intensity is observed in the 4-layer PtSe_2_ sample. The SHG signal intensity of few-layer PtSe_2_ also varies with the number of layers due to the layer-dependent electronic structure and optical properties of PtSe_2_. No obvious SHG signal is detected in the bulk PtSe_2_.

**Figure 4:**
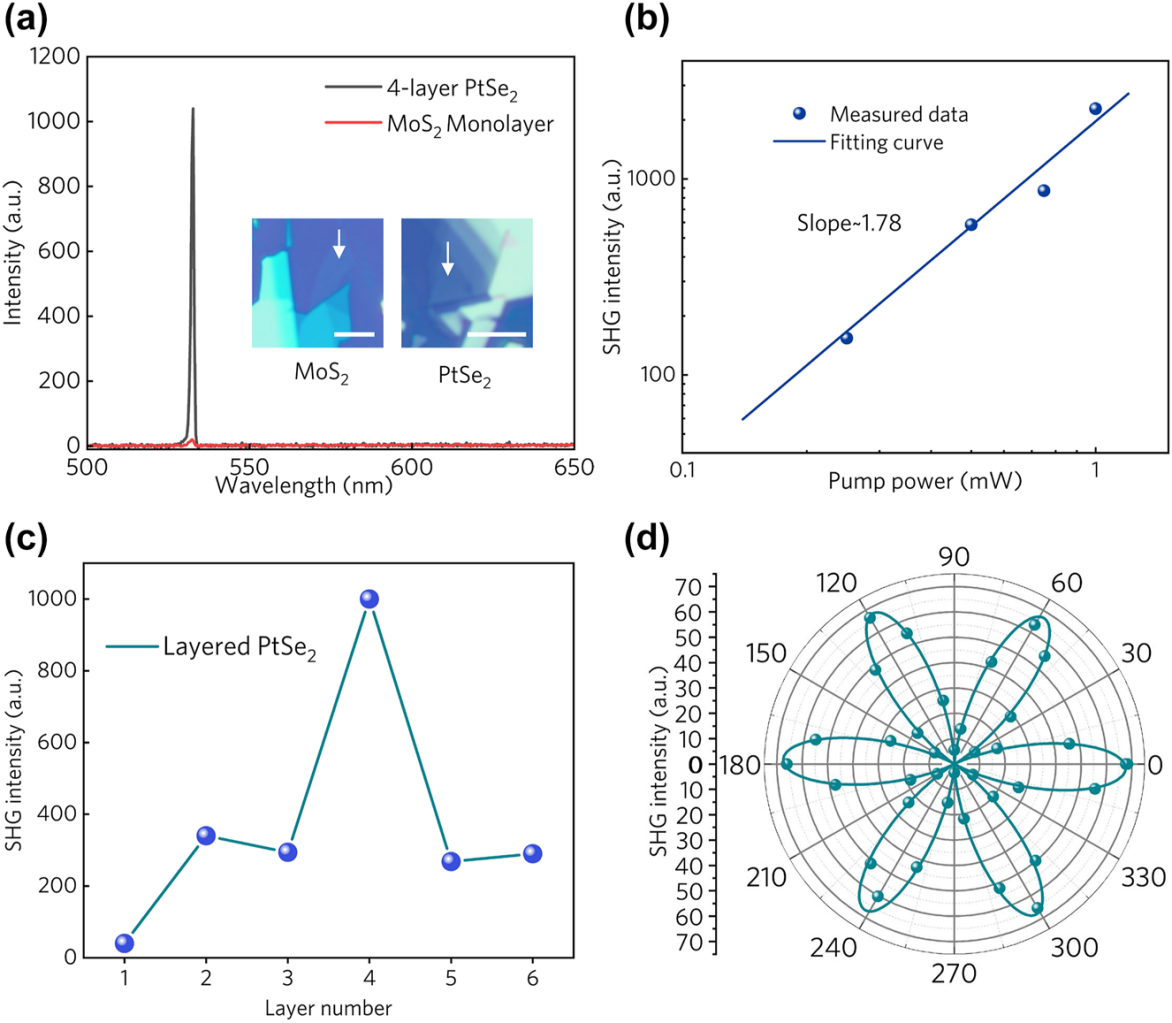
SHG property of two-dimensional PtSe_2_. (a) The SHG intensity comparison of 4-layer PtSe_2_ and MoS_2_ monolayer. Insets are optical microscope images of mechanically exfoliated 4-layer PtSe_2_ and monolayer MoS_2_. (b) The SHG intensity as a function of the pump power in logarithmic coordinates. (c) The SHG intensity with different layer numbers. (d) The polarization-dependent SHG intensity and its fitting curve.

In comparison to mechanically exfoliated monolayer MoS_2_, a well-known traditional strong SHG material [9], [10], the SHG intensity in 4-layer PtSe_2_ excited by a 1064 nm wavelength laser is significantly higher by nearly two orders of magnitude (60-fold), as depicted in Figure 4(a). The test conditions are configured with an input power of 1 mW and an integration time of 5 s. The inset shows an optical microscope image of the tested sample with arrows indicating the test locations. This intense SHG demonstrates PtSe_2_ as a promising candidate for ultrathin nonlinear optical materials in various applications. By examining the SHG intensity maps with varying pump light intensities, we observe a slope close to 2 on logarithmic coordinates (Figure 4(b)), corresponding to the second-order nonlinear process, further confirming the emission of second-harmonic waves. The detailed layer-dependent SHG intensity is depicted in Figure 4(c). The 4-layer PtSe_2_ with enhanced absorption demonstrates increased SHG, whereas with more than 4 layers, the stronger optical absorption in PtSe_2_ impedes a higher SHG efficiency. Thus, the 4-layer PtSe_2_ could serve as a potential candidate for 2D nonlinear optical applications.

Due to the in-plane lattice structure of PtSe_2_, the intensity of its SHG signal also displays polarization-dependent characteristics. As depicted in Figure 4(d), the polarization-dependent SHG intensity in layered PtSe_2_ *I*_SHG_ in 1*T*-type PtSe_2_ can be fitted as [56]

$$I_{\text{SHG}}\propto I_{\max}|\cos\left(3\theta \right){|}^{2},$$

where *I*_max_ refers to the maximum SHG intensity. The polarization-dependent SHG exhibits a six-petal shape, corresponding to the in-plane threefold rotational symmetry of PtSe_2_. Since SHG intensity is highly sensitive to the crystal symmetry and orientation, by measuring the SHG signal with different incident polarization angles, one can extract valuable information about the crystal orientation of PtSe_2_ at a macro scale.

## 3 Conclusions

In summary, the layer-dependent second-order nonlinear optical response in 2D PtSe_2_ has been explored with intriguing results. Intense SHG is prominently observed in few layers of PtSe_2_. Notably, the strongest SHG intensity is found in 4-layer PtSe_2_, surpassing the SHG response of exfoliated MoS_2_ monolayers when excited by a pulsed laser at 1064 nm by nearly two orders of magnitude. Combined with its excellent air-stability, these findings underscore the significant potential of PtSe_2_, especially in its 4-layer form, for ultrathin nonlinear optical applications. The unique layer-dependent SHG behavior of PtSe_2_ opens new avenues for exploring and harnessing its nonlinear optical properties for advanced photonic technologies.

## Supplementary Material

Supplementary Material Details
