# Supplementary material for: Intense second-harmonic generation in two-dimensional PtSe2
Source: Nanophotonics. 2024 Apr 22;13(18):3457–64. doi: 10.1515/nanoph-2024-0107 (PMC11501338; doi:10.1515/nanoph-2024-0107)
Supplement: Supplementary file 1 — Supplementary Material Details [file j_nanoph-2024-0107_suppl_001.pdf]

Supplementary material

Intense second-harmonic generation in two-dimensional $PtSe_2$

Lingrui Chu,[1] Ziqi Li,[2] Han Zhu,[1] Hengyue Lv,[1] and Feng Chen[1 a)]

[1]*School of Physics, State Key Laboratory of Crystal Materials, Shandong University, Jinan 250100, China*

[2]*Division of Physics and Applied Physics, School of Physical and Mathematical Sciences, Nanyang Technological University, Singapore 637371, Singapore*

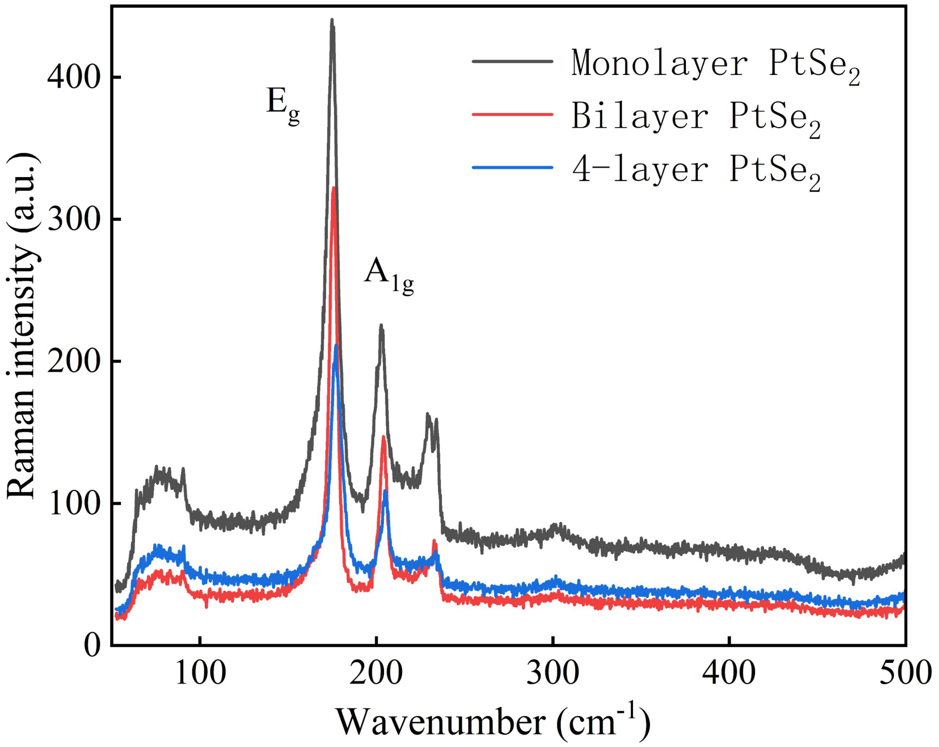

Figure S1: Raman spectra of $PtSe_2$ monolayer, bilayer, and 4-layer $PtSe_2$.

The Raman spectra of layered $PtSe_2$ are measured by a confocal Raman spectrometer (LabRAM HR Evolution). Two distinct peaks at ~175 cm^{-1} and 203 cm^{-1} correspond to E_g and A_{1g} vibrations of 2D $PtSe_2$, respectively. As the number of layers increases, the E_g remains essentially unchanged, while A_{1g} undergoes a slight red shift.

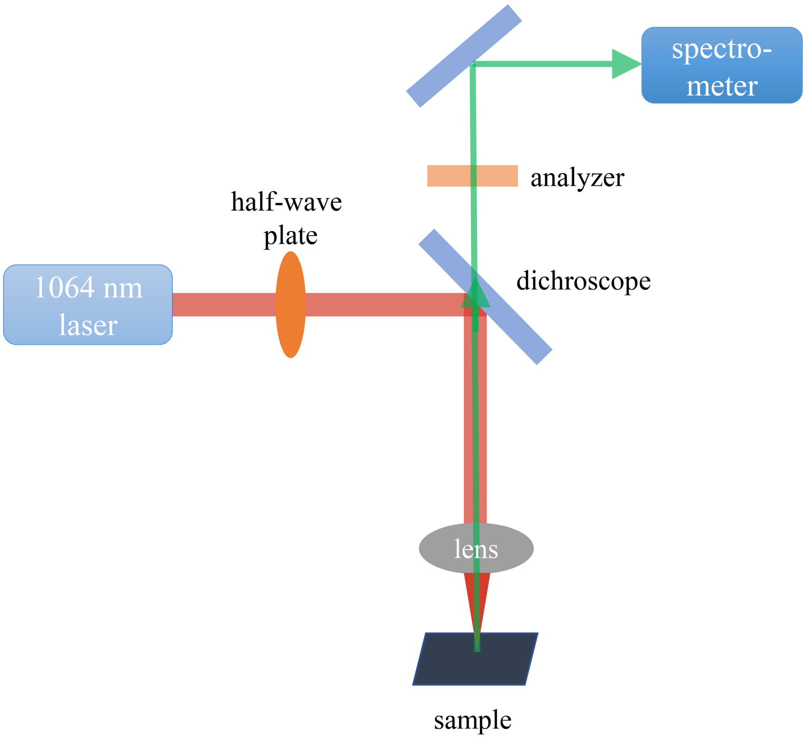

Figure S2. Schematic diagram of SHG measurement setup.
